# Empagliflozin—A New Chance for Patients with Chronic Heart Failure

**DOI:** 10.3390/ph15010047

**Published:** 2021-12-30

**Authors:** Klaudia Kowalska, Justyna Walczak, Joanna Femlak, Ewelina Młynarska, Beata Franczyk, Jacek Rysz

**Affiliations:** Department of Nephrology, Hypertension and Family Medicine, Medical University of Lodz, 90-549 Lodz, Poland; klaudia.kowalska1@stud.umed.lodz.pl (K.K.); justyna.g.walczak@gmail.com (J.W.); femlak.joanna@gmail.com (J.F.); bfranczyk-skora@wp.pl (B.F.); jacek.rysz@umed.lodz.pl (J.R.)

**Keywords:** heart failure, cardiovascular pharmacotherapy, cardioprotection, SGLT2 inhibitors, empagliflozin, cardiovascular diseases, diabetes

## Abstract

The heart failure (HF) epidemic is one of the challenges that has been faced by the healthcare system worldwide for almost 25 years. With an ageing world population and a fast-paced lifestyle that promotes the development of cardiovascular disease, the number of people suffering from heart failure will continue to rise. To improve the treatment regimen and consequently the prognosis and quality of life of heart failure patients, new therapeutic solutions have been introduced, such as an inclusion of Sodium-glucose co-transporter 2 (SGLT-2) inhibitors in a new treatment regimen as announced by the European Society of Cardiology in August 2021. This article focuses on the SGLT2 inhibitor empagliflozin and its use in patients with heart failure. Empagliflozin is a drug originally intended for the treatment of diabetes due to its glycosuric properties, yet its beneficial effects extend beyond lowering glycemia. The pleiotropic effects of the drug include nephroprotection, improving endothelial function, lowering blood pressure and reducing body weight. In this review we discuss the cardioprotective mechanism of the drug in the context of the benefits of empagliflozin use in patients with chronic cardiac insufficiency. Numerous findings confirm that despite its potential limitations, the use of empagliflozin in HF treatment is advantageous and effective.

## 1. Introduction

Heart failure (HF) was named an epidemic for the first time 25 years ago, and its incidence rate has been on the rise ever since [1]. Currently, approximately 64.3 million people in the world are suffering from cardiac insufficiency [1]; its increase in incidence over the past three decades could be assigned to significant changes in the demographics of the world population, HF management, and the incidence and survival of illnesses predisposing to HF [1,2]. Statistics indicate that one person out of five in a developed country is likely to develop the disease during their lifetime [3]. The lifetime risk of HF is 21% for men and 20% for women at the age of 40; moreover, the risk doubles for those whose blood pressure is over 160/100 mmHg, as compared to a group of patients with no history of hypertension [4]. As the world population is ageing and a sedentary lifestyle is more and more popular, the prevalence of HF will continue to grow [5]. Even though HF primarily affects the elderly, the number of young people suffering from it is currently on the rise, whether it be due to the increase in obesity or of diabetes, hypertension and atrial fibrillation amongst the population [1]. Invariably, HF is one of the main causes of hospitalization of adults and remains a significant burden to healthcare all over the world [6].

As for treatment options, since August 2016 there have been three fundamental drug groups on which the treatment regimen for HF with reduced ejection (HFeEF) fraction has been based. The first and particularly beneficial drugs, angiotensin-converting enzyme inhibitors (ACEI), have been proven to improve symptoms, reduce the risk of hospital admission and prolong life. ACEI are typically combined with beta-blockers and aldosterone inhibitors, which seem to have a similar beneficial impact on the patient [7]. Even though this three-drug combination has been considered the gold standard of treating HFrEF, there are many cases when the severity of its symptoms requires additional use of other drugs, such as loop diuretics [5]. As cardiac insufficiency continues to be an increasingly widespread health issue, there has been much research on therapy optimization options. The latest heart failure treatment regimen, published by the European Society of Cardiology (ESC) in August 2021, includes dapagliflozin and empagliflozin, drugs representing sodium glucose co-transporter 2 (SGLT2) inhibitors which until now have been used as diabetes medication, yet have also been proven to have a positive impact on patients with cardiovascular diseases [8,9].

The aim of this review article is to collect previous findings and determine the validity of the use of empagliflozin, the most popular of flozins, in HF treatment, and to discuss its effectiveness as well as the advantages and disadvantages of its use.

## 2. Heart Failure

Pathophysiologically, HF is either an inadequate cardiac output for the organism’s metabolic demands or an adequate cardiac output that is due to neurohormonal compensation, which means the inability of the heart to supply blood to the tissues according to their needs without additional strain [10,11]. The duration and dynamics of this cardiac inadequacy differentiate acute and chronic HF, which have varied temporal courses and treatments [12]. HF is considered heterogeneous not only in its clinical presentation, but also in its etiology. HF syndrome may develop as a result of any structural or functional cardiac disorder. Multiple causes often coexist, yet the worldwide leading factors are hypertension and ischemic heart disease [1]. Apart from vascular factors, smoking, diabetes, obesity, arrhythmias, chronic obstructive pulmonary disease, thyroid pathologies, congenital cardiomyopathies, inflammatory diseases and excessive alcohol intake also predispose individuals to HF syndrome [1,6,13].

According to the ESC guidelines, the essential factors required to establish the diagnosis of HF are: the presence of the symptoms and signs of HF along with objective evidence of cardiac dysfunction in imaging tests—preferably echocardiography—and, in case of any doubt, a favourable response to treatment (Table 1) [8,14]. The main symptoms of cardiac insufficiency are dyspnea, reduced exercise tolerance, fatigue, orthopnoea, nocturnal cough and sleep disturbances. The alarming signs of HF when examining the patient are oedema, pleural effusion, ascites, hepatomegaly, elevated jugular vein pressure, crepitations, wheeze, tachycardia and abnormal heart sounds and murmurs [14]. Diagnostic algorithms indicate a high usefulness of blood natriuretic peptides levels in preliminary HF diagnosis with B-type natriuretic peptide (BNP) level > 35 pg/mL and N-terminal prohormone of B-type natriuretic peptide (NT-pro-BNP) level above 125 pq/mL [8,15]. Survival rate estimates after receiving HF diagnosis are 50% and 10% at 5 and 10 years, respectively [13].

Typical academic classifications divide HF into systolic or diastolic, acute or chronic and left-ventricle or right-ventricle dominant dysfunction. The most popular echocardiographic categorization used during the diagnostic process is one based on the left ventricle ejection fraction (LVEF) value. There are three subtypes of HF that can be differentiated: HF with preserved ejection fraction (HFpEF) with LVEF >50%, HF with mildly reduced ejection fraction (HFmrEF) where the LVEF value is in the range of 40–49% and HFrEF with LVEF below 40% [8]. However, LVEF categorization has been widely criticized due to alleged oversimplification of such a complex and dynamic syndrome as HF [14]. This is an emerging concern because disease phenotypes have evolved since the EF-based categorization was first introduced, and there are other forms of HF that do not necessarily comply with the traditional EF classification, which may lead to misdiagnosis [16]. 

Many clinical classification systems have been proposed to classify severity of HF and guide patient management. The most popular system is the New York Heart Association (NYHA) classification that ranges from essentially asymptomatic patients (NYHA I) to mild (NYHA II, slight limitation in physical activity), moderate (NYHA III, symptoms on light exercise) and severe HF (NYHA IV, breathless at rest) [15]. Clinically, a wide differential diagnostic process is required to establish a specific cause of what a patient presents with considering the fact that more than half of HF patients suffer from a concomitant comorbidity, for example, obesity, diabetes, chronic kidney disease, smoking-induced lung diseases, hypertension and atrial fibrillation [17]. Simultaneously, the conditions mentioned above are proven to have a negative impact on cardiac function, and their presence corresponds with increased severity of HF symptoms and worse prognosis [1,17]. Atypical manifestations of HF are most common in seniors, who often suffer from multiple morbidities, which makes setting the diagnosis challenging and contributes to the delay in implementing proper, cardiac-oriented treatment [15]. Even though HF is considered to be a disease of the elderly, the incident rate in the young has been rising recently. This increase has been linked with poor lifestyle choices, especially those contributing to weight gain as obesity has started to play a prominent role in HF etiology [17]. Surprisingly, obese individuals tend to have a better treatment response. This phenomenon is called the obesity paradox [15].

Therapeutic approach differs depending on HF type. The pharmacological treatment of chronic HFrEF is now based on four classes of drugs that have been proven to reduce mortality among HF patients: ACE inhibitors or angiotensin II receptor blockers (ARBs), beta-blockers, aldosterone antagonists and SGLT2-inhibitors [16]. Adding ivabradine to the scheme may be especially beneficial in patients with a heart rate >70/min in spite of using a beta-blocker in its optimal dose [18]. The mainstay for managing the symptoms of fluid overload is diuretics [16]. In advanced stages, device-based therapies, such as internal cardioverter defibrillator or cardiac resynchronization therapy, may be implemented [16,19]. Lately, a great deal of attention has been drawn to the PARADIGM-HF trial results that proved that a relatively new drug, angiotensin receptor-neprilysin inhibitor [ARNI], has better prognostics, improves the quality of life and reduces hospitalization rate in comparison to the three-drug scheme comprising a beta-blocker, an aldosterone antagonist and enalapril instead of ARNI [13,19,20]. In the new ESC Guidelines, ARNI is recommended as a replacement for ACE-I inhibitors in patients with HFrEF as a Class IB recommendation [8]. There have been promising trials focused on inotropic agents, such as levosimendan, and the oral drug omecamtiv mecarbil [5,18,21]. Moreover, intravenous treatment with iron supplementation in patients with anemia has been shown to improve their quality of life and is associated with a reduction in hospitalization rate [22]. However, oral supplementation does not have such beneficial impact on HF. The therapeutic picture for both HFpEF and HFmrEF is not based on a particular drug scheme; rather, its main focuses are rigorous control of hypertension, optimizing heart rate and reducing fluid retention with diuretics [5]. The pharmacological approach and response to medication may vary depending on the etiology of HF. In every HF subtype, the optimal management of codominant comorbidities, especially diabetes, obesity, and metabolic syndrome, is key in improving clinical outcomes [16]. Pharmacological treatment should also include lipid-lowering, antiplatelet and antithrombotic therapies if recommended. [16] Regardless of the pharmacological approach, the treatment plan should include educating patients about the disease, exercise-based cardiac rehabilitation, smoking cessation, diet modifications and obesity prevention [23,24].

## 3. Empagliflozin as a Representative of SGLT2-Inhibitors

Empagliflozin, approved in 2014, represents a relatively new class of anti-diabetic oral drugs—SGLT2 inhibitors, also known as flozins [25]. In Poland, apart from empagliflozin, there are also two marketed compounds of this group: dapagliflozin and canagliflozin, which are reimbursed for patients with type 2 diabetes approached with combined oral antidiabetic therapy with no satisfactory outcome [26]. In August 2021, dapagliflozin was also approved in the European Union for the treatment of chronic kidney disease regardless of diabetic status, owing to the results of the phase III of A Study to Evaluate the Effect of Dapagliflozin on Renal Outcomes and Cardiovascular Mortality in Patients With Chronic Kidney Disease (DAPA-CKD trial) that showed dapagliflozin delayed disease progression and improved prognosis in individuals with diabetes as well as non-diabetic individuals [25,27]. Chemically, empagliflozin belongs to the C-glucoside family. As an inhibitor, it is competitive and selective and it is additionally quite potent; its role includes glucose reabsorption mediation, which takes place in the proximal tubule of the kidney and leads to the increase in urinary glucose excretion [28]. As a glucose-lowering drug, it is the first that was proven to decrease mortality due to cardiovascular reasons in patients with diabetes mellitus when the Empagliflozin Cardiovascular Outcome Event Trial in Type 2 diabetes Mellitus Patients (EMPA-REG OUTCOME trial) results were published. [29] The promising results of this study aroused the researchers’ interests and provided a starting point to investigate the efficacy of empagliflozin in patients without diabetes, resulting in Empagliflozin Outcome trial in Patients With Chronic Heart Failure With Reduced Ejection Fraction (EMPEROR-Reduced). To this date, empagliflozin is also the first among SGLT2-inhibitors to show beneficial effects in all HF subtypes since the results of the The Empagliflozin Outcome Trial in Patients with Chronic Heart Failure with Preserved Ejection Fraction (EMPEROR-Preserved trial) were published. However, the Dapagliflozin Evaluation to Improve the Lives of Patients With Preserved Ejection Fraction Heart Failure (DELIVER trial) is currently being conducted among patients with LVEF > 40% to investigate the effect of dapagliflozin on the course of HFmrEF and HFpEF.

**Figure 1:**
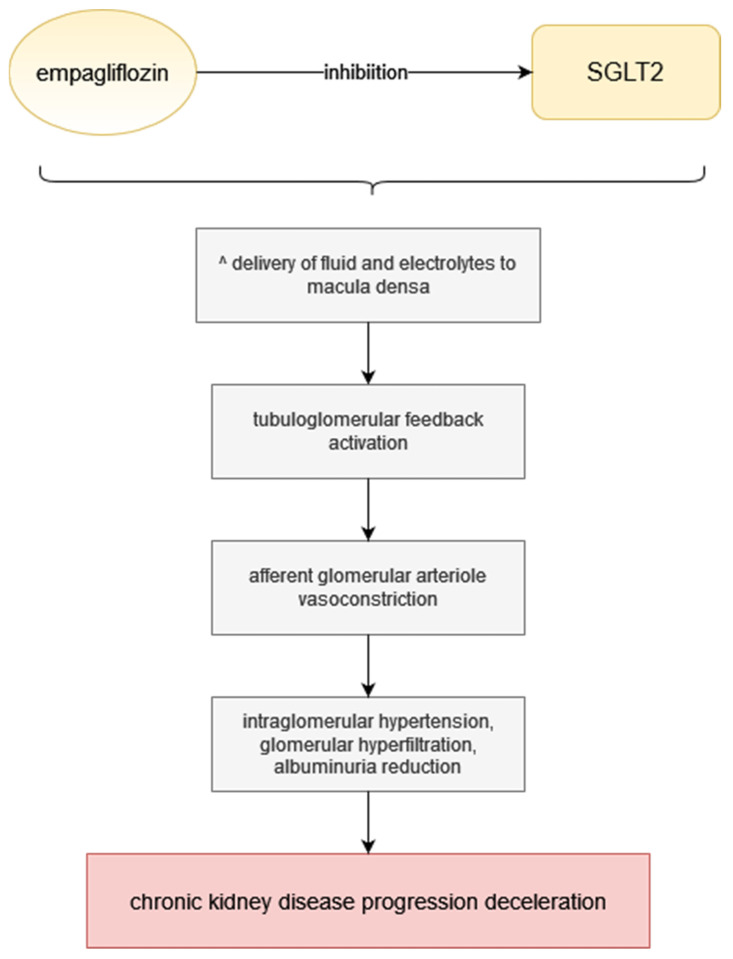
Empagliflozin mechanism of action. Step by step visualisation of action from SGLT inhibition to chronic kidney disease progression deceleration.

Empagliflozin has glucuretic, diuretic and natriuretic properties, which means that through SGLT inhibition, it decreases blood glucose, causes osmotic diuresis and reduces sodium load [29,30]. Approximately 80 g of glucose is excreted every day due to SGLT2 inhibition if the patient maintains normal renal functions [31]. As SGLT2-inhibitors work in the proximal tubules, they increase the delivery of fluid and electrolytes to the macula densa, which activates tubuloglomerular feedback leading to afferent glomerular arteriole vasoconstriction [32,33]. This results in a reduction in intraglomerular hypertension, diminishes glomerular hyperfiltration, attenuates albuminuria and thereby decelerates the progression of diabetic as well as non-diabetic chronic kidney disease (Figure 1) [34,35]. Although a decrease in eGFR may be initially observed during empagliflozin treatment, it is followed by stabilization after a longer period of time [29,36]. Empagliflozin treatment is associated with many beneficial effects, such as weight loss despite increased food intake, largely due to body fat loss, improvement of endothelial dysfunction and arterial stiffness, reduction in blood pressure in diabetic patients and alleviation of the early signs of nephropathy in diabetic animal models; on the other hand, empagliflozin preserved body weight in models of type 1 diabetes [29,31]. Patients with type 2 diabetes were observed to have lost weight and reduced their blood pressure, as well as improved glycemic control after undergoing empagliflozin treatment in monotherapy or as an addition to other medication [27,29]. Other parameters empagliflozin affects positively are HbA1c, waist circumference and uric acid; however, there is no known impact on heart rate or LDL and HDL cholesterol [29].

## 4. The Underlying Mechanism of the Beneficial Effect of Empagliflozin in HF Treatment

**Figure 2:**
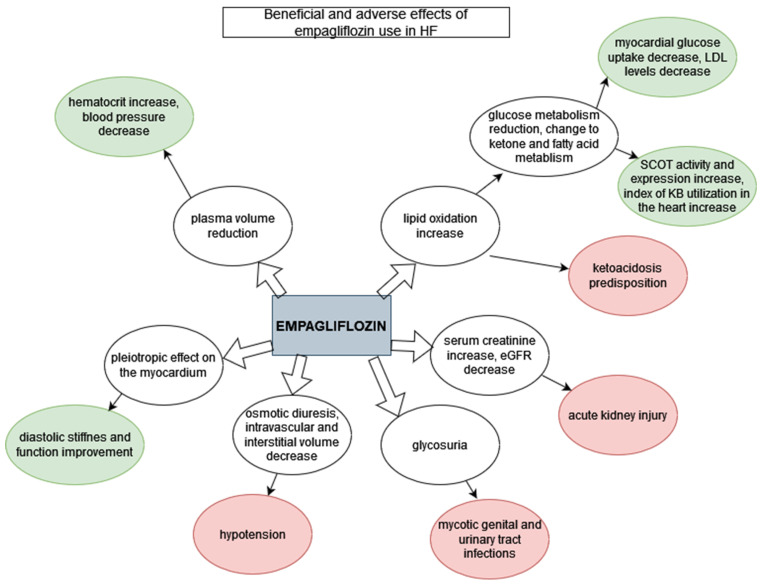
Beneficial and adverse effects of empagliflozin use in HF. Green: beneficial effects; red: adverse effects.

Among the standard comorbidities of diabetes mellitus are artery disease, stroke and most importantly, HF. HF is a major contributor to mortality of patients with type 2 diabetes [31]. There is evidence that empagliflozin given as an adjunct to standard care reduced cardiovascular events, cardiovascular mortality and incidental or worsening nephropathy in patients with type 2 diabetes and established cardiovascular disease [29]; it was also shown to significantly reduce myocardial fibrosis [25]. However, the beneficial effect of empagliflozin on the cardiovascular system is not limited to people with diabetes [8,30,35,37]. Since August 2021, empagliflozin has been placed among the major drugs used in HFrEF treatment scheme, as it reduces the risk of hospitalization and death [8]. Empagliflozin was also proven to be beneficial for patients with HFpEF regardless of codominant diabetes (Figure 2) [37]. 

The mechanism of SGLT2 inhibitor treatment benefit in HF is still yet to be determined; a recent analysis suggested that even though the changes in HbA1c levels achieved by empagliflozin use in patients with diabetes were present, their impact on mortality of cardiovascular causes was moderate, which suggests that the glucose lowering factor was not prevalent [31]. This was confirmed in patients with chronic kidney disease, whose capacity of glucose lowering through empagliflozin use was diminished; however, their cardiovascular mortality was equally lowered [38]. Therefore, there are other theories about the underlying mechanism of empagliflozin’s beneficial influence on patients with HF.

Empagliflozin reduces plasma volume, which seems to be the main aspect influencing mortality rates. This may be observed in the increase in hematocrit [29] and additionally a significant blood pressure decrease with measurements 3 to 4 mmHg lower after 12 weeks [25]. SGLT2 inhibition is hypothesized to result in fluid clearance from the interstitial fluid space rather than circulation through electrolyte-free water clearance, which results in an emphasis on congestion relief as compared to blood volume and organ perfusion [31].

To compensate for a decrease in glucose oxidation in patients taking SGLT-inhibitors, there is an increase in lipid oxidation in order to maintain energy balance [25]; a switch toward ketone and free fatty acid metabolism has been observed after empagliflozin treatment [38]. Reduced myocardial glucose metabolism is evident through lower myocardial glucose uptake and reduced LDL cholesterol levels, while an increase in the uptake of myocardial ketone bodies, increased activity and expression of succinyl-CoA:3-oxoacid CoA-transferase (SCOT) and higher ratio of serum-to-myocardial ketone bodies (an index of ketone body utilization in the heart) show the significance of ketone metabolism [38].

The risk of hospitalization for HF in patients with type 2 diabetes was found to be reduced by 35% with empagliflozin treatment [39]. Empagliflozin also causes pleiotropic effects directly on the myocardium, which improves diastolic stiffness and diastolic function regardless of the underlying diabetes [30]. In another study, there was no influence of empagliflozin on the electrocardiographic parameters observed, nor did it improve LVEF; however it did significantly attenuate cardiac fibrosis of the left atrium and ventricle [25]. Another study showed an amelioration of anatomical remodelling of the left ventricle after myocardial infarction, an improvement of left ventricular systolic function and lower BNP and Troponin I levels with empagliflozin treatment [38].

## 5. Limitations of Empagliflozin Use in Patients with HF

The clinical value of empagliflozin in cardiological patients should be assessed considering not only the potential benefits, but also the adverse effects and risks of using SGLT-2 inhibitors [30]. The use of empagliflozin is not recommended and should not be implemented if Glomerular Filrtation Rate (GFR) is less than 20 mL/min/1.73 m^2^ [40]. GFR-related criterion may often determine limitations in using empagliflozin in patients with HF, as they often suffer from comorbid renal disease [21]. Besides, empagliflozin is not used in patients with HF codominant to type 1 diabetes or in those with diabetic ketoacidosis [41]. Other contraindications include women in their second and third trimesters of pregnancy and those with a severe hypersensitivity reaction to empagliflozin [41].

Empagliflozin has several adverse effects, including hypotension (especially in patients treated with ACE-inhibitors, ARBs or diuretics), acute kidney injury, genital mycotic infections, ketoacidosis, hypoglycemia in patients on insulin, dyslipidemia and extremely rarely, Fournier gangrene and peripheral amputations (Table 2) [40,41]. The adverse events reported with empagliflozin correspond with the specific renal mechanism of action of the SGLT-2 class [42]. Empagliflozin causes osmotic diuresis and reduces both intravascular and interstitial volume; thus it can cause symptomatic hypotension, especially in patients on hypotensive drugs, the elderly, patients with renal impairment and patients with low systolic blood pressure [40,41,42]. SGLT2-inhibitors may interact with loop diuretics commonly used in patients with HF; thus, an adjustment of doses is required [19]. Additionally, withdrawal of empagliflozin may be necessary for patients with clinical hypovolemia or more severe adverse effects, such as ketoacidosis [19]. Hospitalization for surgery or any acute medical conditions predisposes patients to euglycemic ketoacidosis, which has been reported more often during treatment with SGLT-2 inhibitors and requires ketoacidosis prevention in the form of changing from oral treatment to insulin injections [40]. Apart from reducing volemia and predisposing individuals to ketoacidosis, empagliflozin increases serum creatinine and decreases eGFR, which may result in acute kidney injury. When implementing SGLT-2 inhibitors, renal function should be initially evaluated and regularly monitored [41]. Mycotic genital infections and urinary tract infections are common in the context of treatment with SGLT2-inhibitors [43] and appear to be troublesome for patients; they may lead to the discontinuation of treatment. However, they can be prevented by meticulous hygiene [41]. Patients started on empagliflozin therapy should be informed of the risk of those complications, instructed about preventing them, regularly evaluated for signs and symptoms and treated properly if they occur [19,41].

## 6. Empagliflozin versus Dapagliflozin in HF Treatment

Both dapagliflozin and empagliflozin are listed in the ESC Guidelines for Diagnosis and Treatment of Acute and Chronic Heart Failure. In Table 3, we compared the most relevant studies on the use of these two drugs for heart failure. The primary endpoints in each of these studies are death from cardiovascular causes or worsening of heart failure treated as urgent hospitalization or an event requiring intravenous therapy. The report of the DELIVER study, which is investigating the effect of dapagliflozin on HFmrEF and HFpEF and is complementary to the DAPA-HF study, is not yet available. DELIVER is also equivalent in some ways to the EMPEROR-Preserved study involving empagliflozin. The DAPA-HF, EMPEROR-Reduced and EMPEROR-Preserved studies showed significant effects of both dapagliflozin and empagliflozin in reducing mortality and the number of hospitalizations compared to the placebo group. The effects were consistent across prespecified subgroups, including the presence or absence of diabetes at baseline. To date, empagliflozin remains the only SGLT2 inhibitor whose efficacy extends over a wide range of LVEF, as proven in the EMPEROR-Preserved study; therefore, it has advantages over dapagliflozin.

Meta-analyses comparing the DAPA-HF and EMPEROR-Reduced trials highlight the differences in enrolment criteria regarding NT-pro-BNP value, with a conclusion that the EMPEROR-Reduced trial targeted more advanced HF patients [45]. In the EMPEROR-Reduced trial, the baseline median LVEF and eGFR were lower and the median NT-pro-BNP was higher than in DAPA-HF patients [45]. These differences in patient selection are reflected in a higher number of events in the placebo group and a higher percentage of cardiovascular deaths or hospitalizations in the empagliflozin trial. However, there were fewer HF hospitalizations over 12 months of follow-up in patients treated with empagliflozin [45]. In both trials, the percentage of patients with diabetes in each group was similar and all patients received properly tailored HF therapy. Both the DAPA-HF and EMPEROR-Reduced trials demonstrated consistent outcomes of cardiovascular death or heart failure hospitalizations, with around a 25% reduction in the groups assigned to the SGLT2-inhibitors [45]. Both dapagliflozin and empagliflozin showed a similar and significant effect in attenuating the decline in eGFR, therefore demonstrating effective renoprotection. Analyses combining the results of both studies involving 8474 patients estimated the effect of SGLT-2 treatment as a 13% reduction in all causes of death, 14% reduction in cardiovascular death, 26% reduction in the combined risk of cardiovascular death and first hospitalization for HF and 25% decrease in recurrent hospitalizations for HF and cardiovascular death [46]. To conclude, the benefits of implementing SGLT-2 inhibitors in the treatment of heart failure are indisputable and the efficacy of empagliflozin and dapagliflozin are similar. More studies and prospective head-to-head comparisons are needed to conclusively determine which SGLT-2 inhibitor is a better choice.

**Table 3:** Comparison of double-blind, randomized clinical trials on dapagliflozin and empagliflozin in Heart failure (HF).

Selected Clinical Trials on SGLT-2 Inhibitors in Heart Failure				
FLOZIN	DAPAGLIFLOZIN		EMPAGLIFLOZIN	
STUDY	DAPA-HF [47]	DELIEVER [48]	EMPEROR-Reduced [44]	EMPEROR-Preserved [49]
INCLUSION CRITERIA	Patients of an age of at least 18 years, an ejection fraction of 40% or less, NYHA class II, III, or IV. Patients were required to have a plasma level of N-terminal pro–B-type natriuretic peptide (NT-proBNP) of at least 600 pg per millilitre (or ≥400 pg per millilitre if they had been hospitalized for heart failure within the previous 12 months). Patients with atrial fibrillation or atrial flutter on baseline electrocardiography were required to have an NT-proBNP level of at least 900 pg per millilitre.	Patients at 40 years of age or older, with an LVEF > 40%, evidence of structural heart disease and elevation in natriuretic peptides [N-terminal pro B-type natriuretic peptide (NT-proBNP) ≥300 pg/mL (≥600 pg/mL for patients in atrial fibrillation or flutter).	Adults (≥18 years of age) who had chronic heart failure (functional class II, III, or IV) and - LVEF ≤30% and NT-proBNP ≥600 pg/mL (without AF) and ≥1200 pg/mL (with AF); - LVEF 31–35% and NT-proBNP ≥1000 pg/mL (without AF) and ≥2000 pg/mL (with AF); - LVEF 36–40% and NT-proBNP ≥2500 pg/mL (without AF) and ≥5000 pg/mL (with AF); - LVEF >40% and hospitalization for heart failure in past 12 mo and NT-proBNP ≥600 pg/mL (without AF) and ≥1200 pg/mL (with AF)	Patients with LVEF >40%, elevated N-terminal pro B-type natriuretic peptide (NT-proBNP) concentrations (i.e., >300 pg/mL in patients without and >900 pg/mL in patients with atrial fibrillation) along with evidence of structural changes in the heart or documented history of heart failure hospitalization. NYHA II-IV.
NUMBER OF PATIENTS	4744	6263	3730	5988
DIABETIC STATUS	Both diabetic and non-diabetic patients.	Both diabetic and non-diabetic patients.	Both diabetic and non-diabetic patients.	Both diabetic and non-diabetic patients.
MEAN FOLLOW-UP TIME	18.2 months.	Ongoing trial.	16 months.	26.2 months
PRIMARY ENDPOINT	Worsening heart failure (urgent hospitalization or intravenous therapy) or death from cardiovascular causes.	Cardiovascular death or worsening heart failure event (heart failure hospitalization or urgent HF visit).	Adjudicated cardiovascular death or hospitalization for heart failure, analysed as the time to the first event.	A composite of adjudicated cardiovascular (CV) death or hospitalization for HF.
PRIMARY ENDPOINT OCCURENCE	The primary outcome occurred in 386 of 2368 patients (16.3%) in the dapagliflozin group and in 502 of 2375 patients (21.2%) in the placebo group (HR: 0.74).	Ongoing trial	Primary outcome event occurred in 361 of 1863 patients (19.4%) in the empagliflozin group and in 462 of 1867 patients (24.7%) in the placebo group (hazard ratio for cardiovascular death or hospitalization for heart failure: 0.75).	A primary composite outcome event occurred in 415 patients (13.8%) in the empagliflozin group and in 511 patients (17.1%) in the placebo group, HR: 0.79.
DIABETIC VS. NON-DIABETIC GROUP	Findings in patients with diabetes were similar to those in patients without diabetes.	Ongoing trial	The effect of empagliflozin on the primary outcome was consistent in patients regardless of the presence or absence of diabetes.	The effects were consistent across prespecified subgroups, including in the presence or absence of diabetes at baseline.

## 7. Conclusions

With the changes made to the HF treatment guidelines in August 2021, empagliflozin has become a new mainstay of HF management. Numerous findings confirm that the use of empagliflozin in HF is both advantageous and effective. The most informative clinical trials that have been conducted on empagliflozin in heart failure determining its effectiveness and safety are EMPEROR-Reduced and EMPEROR-Preserved, both comparing the efficacy of empagliflozin in patients regardless of their diabetic status, and EMPA-REG OUTCOME, focused on patients with diabetes. These studies have demonstrated that empagliflozin significantly improves cardiovascular outcomes, reduces hospitalization rates and lowers the risk of death in patients with heart failure and reduced ejection fraction both with and without diabetes. Due to the lack of standardized pharmacological therapy dedicated to HFpEF patients, the results of the EMPEROR-Preserved trial are particularly promising for this group of patients, as current methods of managing the disease are limited to treating risk factors and reducing symptoms, not improving prognosis. Our review has highlighted the newest research on the mechanism of action, benefits and limitations of empagliflozin, used both as a diabetes mellitus medication and, more importantly, a way of treating HF. We also compared the results of the empagliflozin clinical trials with studies on dapagliflozin, and showed empagliflozin to be more advantageous due to its efficacy in each type of heart failure. The most significant and clinically desirable effects of empagliflozin appear to be, alongside the reduction in blood glucose levels, the induction of osmotic diuresis and the reduction in sodium load and, as a consequence of the above-mentioned, the reduction in plasma volume. The multifaceted effect of the drug is particularly beneficial given the profile of patients with HF. People diagnosed with HF often have a complex medical history with multiple medical conditions, most commonly diabetes and renal failure. Some of these comorbidities can be both a cause and a complication of heart failure; therefore, a drug with such broad pleiotropic properties that has the potential to delay the moment of overlap between these disorders seems particularly beneficial. Introducing SGLT-2 inhibitors to the HF drug scheme is a promising way to optimize the management of the disease and improve not only the lifespan of HF patients, but, more importantly, their quality of life. When the implementation of empagliflozin in treatment is being considered, the contraindications to its use should be kept in mind. The main contraindications include severe renal insufficiency, the second and third trimester of pregnancy and type 1 diabetes. The possibility of developing hypotension in HF patients treated with ACE-inhibitors and diuretics should also be taken into account, especially in those with low blood pressure at the beginning of the treatment. In order to avoid self-discontinuation of the drug, the patient should be informed on the side effects of empagliflozin in the form of genitourinary infections and receive instructions on how to prevent them. Consequently, there is a growing need for research on other flozins, such as canagliflozin and ertugliflozin, in order to determine their effectiveness in HF treatment, as well as the advantages and disadvantages of their use in the context of the contraindications and adverse effects they promote. There is also a strong need for prospective comparisons of empagliflozin and dapagliflozin to clearly determine which SGLT-2 inhibitor is a better choice for the treatment of HFrEF.

## Figures and Tables

**Table 1:** Determination of Heart failure (HF) phenotype according to ESC 2021 Guidelines.

	HFrEF	HFmrEF	HFpEF
**Criterion number 1**	Symptoms and signs	Symptoms and signs	Symptoms and signs
**Criterion number 2: LVEF**	<40%	40–49%	>50%
**Criterion number 3**	-	-	Objective evidence of cardiac structural and/or functional anomalies

**Table 2:** Prevalence of major side effects of empagliflozin in the study and placebo groups.

Adverse Effect	Anker SD et al. [44]		Zinman et al. [43]	
	Empagliflozin Group	Placebo	Empagliflozin Group	Placebo
**Hypotension**	176/1863	163/1863	-	-
	9.45%	8.75%		
**Confirmed hypoglycemia**	33/1863	35/1863	1303/4684	650/2333
	1.77%	1.88%	27.80%	27.80%
**Urinary tract infection**	91/1863	83/1863	842/4684	423/2333
	4.88%	4.46%	17.97%	18.12%
**Acute renal failure**	175/1863	192/1863	246/4684	155/2333
	9.39%	10.3%	5.25%	6.64%

## Data Availability

Data is contained within the article.
